# Metabolic profiles of peanut (*Arachis hypogaea* L.) in response to *Puccinia arachidis* fungal infection

**DOI:** 10.1186/s12864-023-09725-3

**Published:** 2023-10-23

**Authors:** Visha Rathod, Khyati Rathod, Rukam S. Tomar, Ritisha Tatamiya, Rasmieh Hamid, Feba Jacob, Nasreen Shakil Munshi

**Affiliations:** 1 Institute of Science, Nirma University, Ahmedabad, Gujarat India; 2 Department of Biotechnology and Biochemistry, Junagadh Agricultural University, Junagadh, Gujarat India; 3 Department of Bioscience, Saurashtra University, Rajkot, India; 4 Department of Plant Breeding, Cotton Research Institute of Iran (CRII), Agricultural Research, Education and Extension Organization (AREEO), Gorgan, Iran; 5 Centre for Plant Biotechnology and Molecular Biology, Kerala Agricultural University, Thrissur, India

**Keywords:** GC-MS, *Arachis hypogaea*, *Puccinia Arachidis*, Metabolomics, pathogen Infection, Phenolics

## Abstract

*Background Puccinia arachidis* fungus causes rust disease in the peanut plants (*Arachis hypogaea* L.), which leads to high yield loss. Metabolomic profiling of *Arachis hypogaea* was performed to identify the pathogen-induced production of metabolites involved in the defense mechanism of peanut plants. In this study, two peanut genotypes, one susceptible (JL-24) and one resistant (GPBD-4) were inoculated with *Puccinia arachidis* fungal pathogen. The metabolic response was assessed at the control stage (0 day without inoculation), 2 DAI (Day after inoculation), 4 DAI and 6 DAI by Gas Chromatography-Mass Spectrometry (GC-MS). Results About 61 metabolites were identified by NIST library, comprising sugars, phenols, fatty acids, carboxylic acids and sugar alcohols. Sugars and fatty acids were predominant in leaf extracts compared to other metabolites. Concentration of different metabolites such as salicylic acid, mannitol, flavonoid, 9,12-octadecadienoic acid, linolenic acid and glucopyranoside were higher in resistant genotype than in susceptible genotype during infection. Systemic acquired resistance (SAR) and hypersensitive reaction (HR) components such as oxalic acid was elevated in resistant genotype during pathogen infection. Partial least square-discriminant analysis (PLS-DA) was applied to GC-MS data for revealing metabolites profile between resistant and susceptible genotype during infection. Conclusion The phenol content and oxidative enzyme activity i.e. catalase, peroxidase and polyphenol oxidase were found to be very high at 4 DAI in resistant genotype (p-value < 0.01). This metabolic approach provides information about bioactive plant metabolites and their application in crop protection and marker-assisted plant breeding.

**Supplementary Information:**

The online version contains supplementary material available at 10.1186/s12864-023-09725-3.

## Introduction

Peanut (*Arachis hypogaea* L.) is one of the major oilseed crops. Moreover, it is a major source of protein, vitamins and bioactive materials [1]. It is grown in the semi-arid tropical regions of the world. India is the second-highest producer of peanut worldwide, contributing 6.8 million tons by cultivation from 5.8 million ha of its agricultural land in 2017 (faostat.fao.org) [2]. The global production of groundnut is severely affected where about 50 to 70% yield loss occurs due to various foliar fungal diseases, such as rust, late leaf spot (LLS) and early leaf spot (ELS) [3]. Late leaf spot and early leaf spot are leaf diseases caused by *Phaeoisariopsis personata* and *Cercospora arachidicola* respectively. They cause leaf spot and lesion on leaves, leading to defoliation in peanut plant. The basidiomycete fungus *Puccinia arachidis* causes the rust disease in peanut. It has been observed that without chemical control measures, there is a severe prevalence of disease (up to 57% economic damage to the crop) or even destruction of the crop [4].

Plants have evolved various defense mechanisms such as the accumulation of reactive oxygen radicals and the production of phenolic compounds and antimicrobial substances to protect themselves from pathogens [5]. These compounds are formed during the primary and secondary metabolism, and the induction of their production is triggered when a pathogen-associated molecular pattern (PAMP) is detected. This can lead to the production of phytoalexins such as flavonoids, which prevent the growth of the pathogen by disrupting its metabolism and cell structure [6]. Metabolite profiling to determine plant responses to biotic/abiotic stresses has been recommended as an extremely valuable and well-established method [7]. Numerous techniques have been used to study metabolomics, including nuclear magnetic resonance (NMR) spectroscopy, Liquid Chromatography-Mass Spectrometry (LC-MS) and Gas Chromatography-Mass Spectrometry (GC-MS) [8].

A variety of plant-pathogen interactions have been successfully understood using metabolomics approaches. For instance, resistance metabolic profiling of the wheat plant (sumai3-resistant genotype) was studied for resistance against *Fusarium graminearum* pathogen causing considerable losses in yield and grain quality [9]. The metabolite profile of rice plants was studied during infection with *Magnaporthe grisea* the causal agent of rice blast, using the GC-MS technique [10]. Pathogen-induced metabolites such as phenylpropanoids and a flavonoid were identified in tomato plants by metabolomics studies. Nuclear magnetic resonance (NMR) spectroscopy in combination with multivariate data analysis were performed to analyse metabolic changes during plant–pathogen interaction [11]. Transcriptional and metabolic responses were studied in *Brachypodium distachyon* plant during fungal infection caused by *Fusarium graminearum*, which leads to diseases in diverse organs of the Plant. Transcription factors and defense-related metabolites were significantly upregulated during infection by the pathogen [12].The metabolites associated with resistance in potato (*Solanum tuberosum*) were identified by non-target metabolomic profiling during infection with *Phytophthora infestans* [13]. To date, only a few reports on metabolomics in peanut plants have been published. Different metabolites were analyzed in peanut genotypes at different temperature regimes, i.e., low (early sowing date), normal (normal sowing date) and high temperature (late sowing date) under field conditions [14]. Metabolic profiles of *Arachis hypogaea* genotypes were studied during biotic stress by *Sclerotium rolfsii* fungi [15]. However, there is no metabolic study on the effects of rust pathogens on peanut plants has been conducted so far.

The metabolome of higher plants consists of thousands of primary and secondary metabolites, of which almost 10% have been identified so far and many of which play an important role in the plant’s defense system [16]. Metabolomic profiling can identify bio-markers for marker-assisted selection, variety selection and plant protection in plant breeding. The physiological status of the plant and its responses to various biotic and abiotic stimuli are reflected in the quantitative and qualitative composition of the plant metabolome, which thus serves as the linkage between genotypes and phenotypes.

The aim of this study was to understand the metabolome profile of peanut during *Puccinia arachidis* infection. It was performed by GC–MS, which contributes to the understanding of the up-regulation and down-regulation of peanut metabolites during *Puccinia arachidis* infection. Further, it may help to identify metabolite markers which may aid in differentiating rust-resistant and susceptible peanut genotypes.

## Materials and methods

### Plant material and inoculation procedure

Leaf samples of resistant (GPBD-4) and susceptible (JL-24) genotypes of *Arachis hypogaea* were used for the present study. Genotype selection was based on the degree of resistance to rust disease [17]. For inoculation, spore concentrations were measured with a hemocytometer and adjusted to 10^5^ spores/ml with sterilized distilled water and Tween 80 (Sigma-Aldrich, MO, USA) and sprayed on 40-day old plants of the resistant and susceptible genotypes of *A. hypogaea.* The plots were irrigated and optimal growth conditions for *A. hypogaea* were maintained viz. high humidity and temperature at ~ 25˚C, by covering the plots with a thin plastic sheet [18]. The control treatments were carried out under similar conditions without any inoculation.

**Fig. 1:**
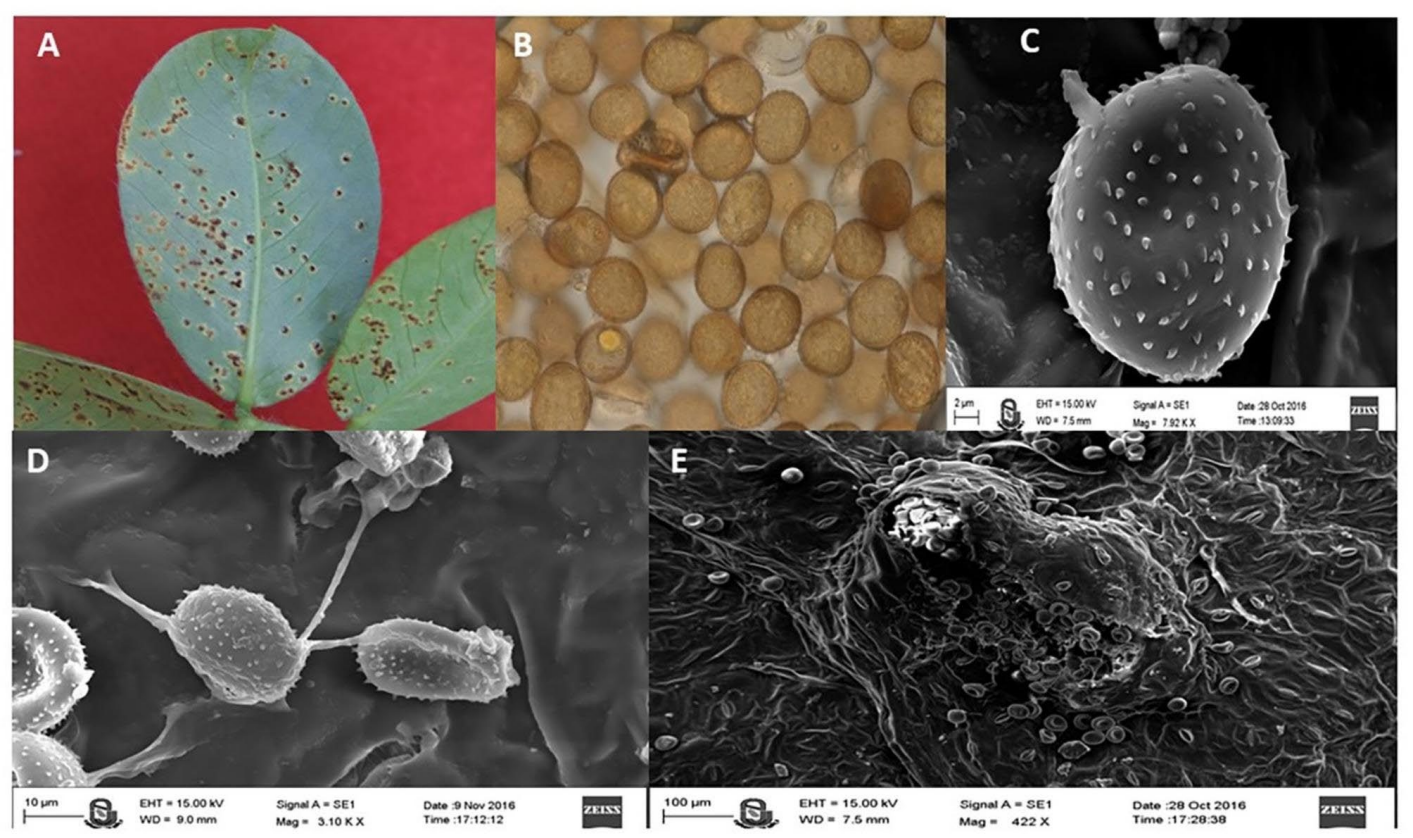
Morphological and electron microscopic study of peanut leaves infected with *Puccinia arachidis* fungi. (**A**. *Puccinia arachidis* sporulation on peanut leaves, **B**. Uredospore of *Puccinia arachidis* fungi, **C**. Electron microscopy of *P. arachidis* spore, D. germ tube and appressorium structure formation from the spore, **E**. Infection by *Puccinia arachidis* spores and insertion in peanut leaf)

Leaves of both the genotypes were collected in three biological replicates at four different stages, i.e., control stage (0 Day - before inoculation), infection stage 1 (2 Days After Infection, i.e. 2 DAI), infection stage 2 (4 DAI) and infection stage 3 (6 DAI). All samples were collected and rapidly frozen in liquid nitrogen. These samples were stored in deep freeze at -80 °C for further experiments. To study metabolism profile, all samples were collected in triplicate. Simultaneously, a microscopic study was carried out to study the morphology of the pathogen and to investigate the release of uredospore which is necessary for confirmation of pathogenic activity (Fig. 1). The sections were dried and treated for gold ionization using ion sputter. The images were taken using the ZEISS-EVO-18 special edition of a scanning electron microscope. Seeds were collected from DGR (directorate of ground research center. The collection of seeds and the complete experiment was carried out according to the national guidelines [19].

### Sampling, metabolite extraction and derivatization

Metabolomics of plant samples were performed with a slight modification in protocol described by Lisec [20]. For extraction, 100 mg of plant leaves (for each sample) were finely powdered in liquid nitrogen using a mortar and pestle, 1.4 ml of methanol was added, vortex-mixed and incubated at 70 °C for 15 min. As an internal quantitative standard, 100 µl of Ribitol (stock of 0.2 mg/ml in dH_2_O) was used. Then 1 ml of Milli-Q followed by 0.75 ml of chloroform (HPLC grade) (Sigma-Aldrich, MO, USA) were added to the sample extract. This methanol-water-chloroform mixture was used for the extraction of hydrophilic and hydrophobic compounds by a single method. Two separate phases of samples viz. polar as well as non-polar were obtained by centrifuging at 10,000 rpm for 10 min. The fractions were dried completely under the nitrogen stream using a turbo-evaporator (Athena Technology, Maharashtra, India).

The derivatization of the metabolites was performed as per the protocol described by Sanimah et al., (2013) with a slight modification [21]. Methoxylation was performed to protect carbonyl moieties by the addition of 100 µl methoxylamine hydrochloride solutions (Sigma-Aldrich, MO, USA) (20 mg/ml in pyridine) and incubated for 90 min at 37 °C in an orbital shaker. In the following step, 100 µl of MSTFA (N-methyl-N-trimethylsilyl trifluoroacetamide) (Sigma-Aldrich, MO, USA) was added and incubated at 37 °C for 60 min and at 110 rpm for silylation.

### Gas chromatography-mass spectrometry (GC-MS) analysis

One hundred µl of these derivatized samples were added to micro inserters, which were then kept in the autosampler vials (2 ml). The entire scan mass spectra for the mass ranging from 50 to 1000 Da was acquired at the scan rate of 0.5 s^− 1^ and a solvent delay out time of 10 min. one µl of each sample was injected using a split ratio of 10:1 into DB-17 MS capillary column (50% phenyl)-methylpolysiloxane (30 m length, 0.25 mm I.D., 0.25 μm film thickness; Agilent Technologies Inc.) equipped with Shimadzu GC-2010 coupled with MS-QP2010 Plus. Electron ionization of 70 eV was used and helium as a carrier gas with a constant flow rate of 1 ml min^− 1^ was maintained. The temperatures for the ion source, the transfer line and the injector were set to 230 °C, 280 °C, and 280 °C respectively. Initially, the oven temperature was kept at 100 °C for 5 min and subsequently increased up to 290 °C at the rate of 5 °C min^− 1^. The mass spectra of the metabolites were matched with the spectra available in the NIST 14 library (National Institute of Standards and Technology, Gaithersburg, MD, USA) for their putative identification. ACD/Spec Manager v.12.00 (Advanced Chemistry Development, Inc., ACD/Labs, Toronto, Canada) was used for various pre-processing steps of Total Ion Chromatograms (TIC) such as baseline correction, alignment, peak picking, and integration. For further data, analysis, CSV comma-delimited files were used [22].

### Metabolomic data analysis

The untargeted metabolite data was processed and the online statistical software MetaboAnalyst 4.0 (https://www.metaboanalyst.ca/) and SPSS software were used for statistical analysis [23, 24]. The peak areas of the chromatograms were considered for statistical analyses. Ribitol was used as an internal standard to normalize the data. Samples were analyzed mainly using the partial least squares-discriminant analysis (PLS-DA) and principal components analysis (PCA). PLS-DA, a frequently used method for analyzing enormous metabolomics datasets is capable of assessing linear/polynomial correlation among variable matrices [14, 25]. PLS-DA was employed to identify important metabolites and the outliers at each stage [15, 26]. The relative levels and relationships of metabolites were visualized using a heat map and dendrogram analysis [24]. Also, data were normalized for untargeted metabolomic analysis with pareto scaling. MetPA (Metabolomic Pathway Analysis) web-based tool was used to perform pathway analysis. Total identified metabolites were submitted into MetPA with common chemical names. All accepted metabolites were verified manually using KEGG and PubChem.

### Enzyme assay

#### Extraction procedure of enzymes

One g of leaf sample was crushed in 10 ml of extraction buffer containing 0.1 M phosphate buffer and 0.5 mM EDTA (pH 7.5) followed by centrifugation using Beckman Coulter Allergera™X-22R centrifuge for 20 min at 15,000 rpm. The supernatant was collected and utilized for the enzyme assays. For the Catalase enzyme assay, 1500 µl of 100 mM phosphate buffer of pH 7.0, 500 µl of 75 mM H_2_O_2_, 500 µl of milli-Q and enzyme supernatant was taken in the quartz cuvette and the OD values were recorded at 240 nm for one min, at intervals of 15 s in the spectrophotometer. The OD values for Peroxidase enzyme assay were recorded by taking 1000 µl of 100 mM phosphate buffer of pH 6.1, 500 µl of 96 mM Guaiacol, 400 µl of milli-Q, 500 µl of 12 mM H_2_O_2_ and 100 µl enzyme supernatant in the quartz cuvette at 470 nm for one min, at intervals of 15 s in the spectrophotometer. Similarly, the OD values for Polyphenol oxidase enzyme assay were measured by taking 2900 µl of 0.1 M catechol in the 10 mM phosphate buffer of pH 6.0, 100 µl milli-Q and enzyme supernatant in the quartz cuvette at 490 nm for one min, at intervals of 15 s in the spectrophotometer. The OD value for a similarly treated control sample (blank without enzyme supernatant) was deducted from test readings [27].

#### The specific activity of enzymes was calculated by

Specific activity (U mg^− 1^ of protein) =

$$\frac{\text{C}\text{h}\text{a}\text{n}\text{g}\text{e} \text{i}\text{n} \text{O}\text{D} \text{p}\text{e}\text{r} \text{m}\text{i}\text{n}\text{u}\text{t}\text{e} }{\text{m}\text{o}\text{l}\text{a}\text{r} \text{e}\text{x}\text{t}\text{i}\text{n}\text{c}\text{t}\text{i}\text{o}\text{n} \text{c}\text{o}\text{e}\text{f}\text{f}\text{i}\text{c}\text{i}\text{e}\text{n}\text{t} \text{o}\text{f} \text{e}\text{n}\text{z}\text{y}\text{m}\text{e} \times \text{v}\text{o}\text{l}\text{u}\text{m}\text{e} \text{o}\text{f} \text{e}\text{n}\text{z}\text{y}\text{m}\text{e} \text{i}\text{n} \text{s}\text{a}\text{m}\text{p}\text{l}\text{e}}\times \text{T}\text{o}\text{t}\text{a}\text{l} \text{p}\text{r}\text{o}\text{t}\text{e}\text{i}\text{n} \text{c}\text{o}\text{n}\text{t}\text{e}\text{n}\text{t}$$

Where,

The molar extinction coefficient of enzyme are as follows:

Catalase = 39.4 U/µmols/g of fresh weight.

Peroxidase = 26.6 U/µmols/g of fresh weight.

Polyphenol oxidase = 1.0 U/µmols/g of fresh weight.

## Results

### Untargeted metabolic profiling by GC-MS analysis

**Fig. 2:**
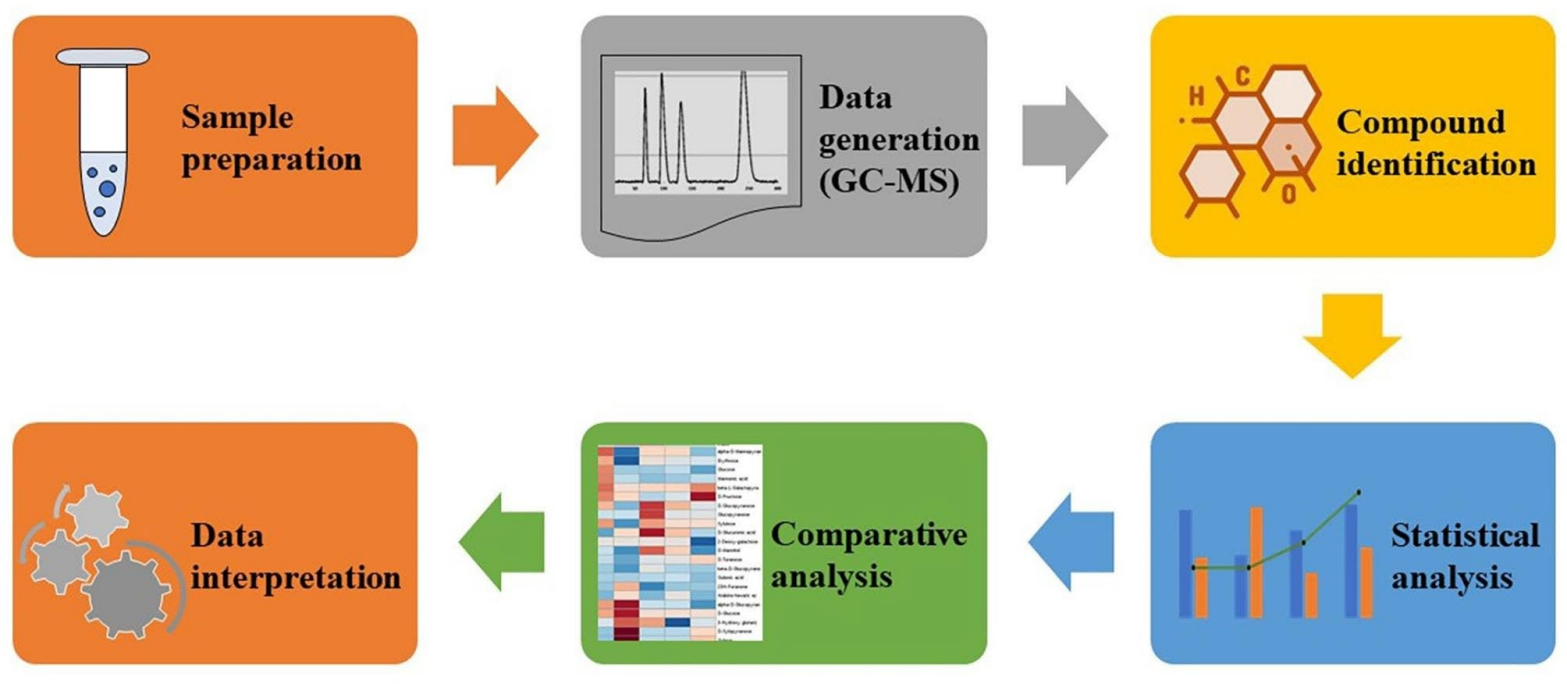
A detailed workflow performed for the metabolome profiling in resistant and susceptible genotypes of *Arachis hypogaea*

**Fig. 3:**
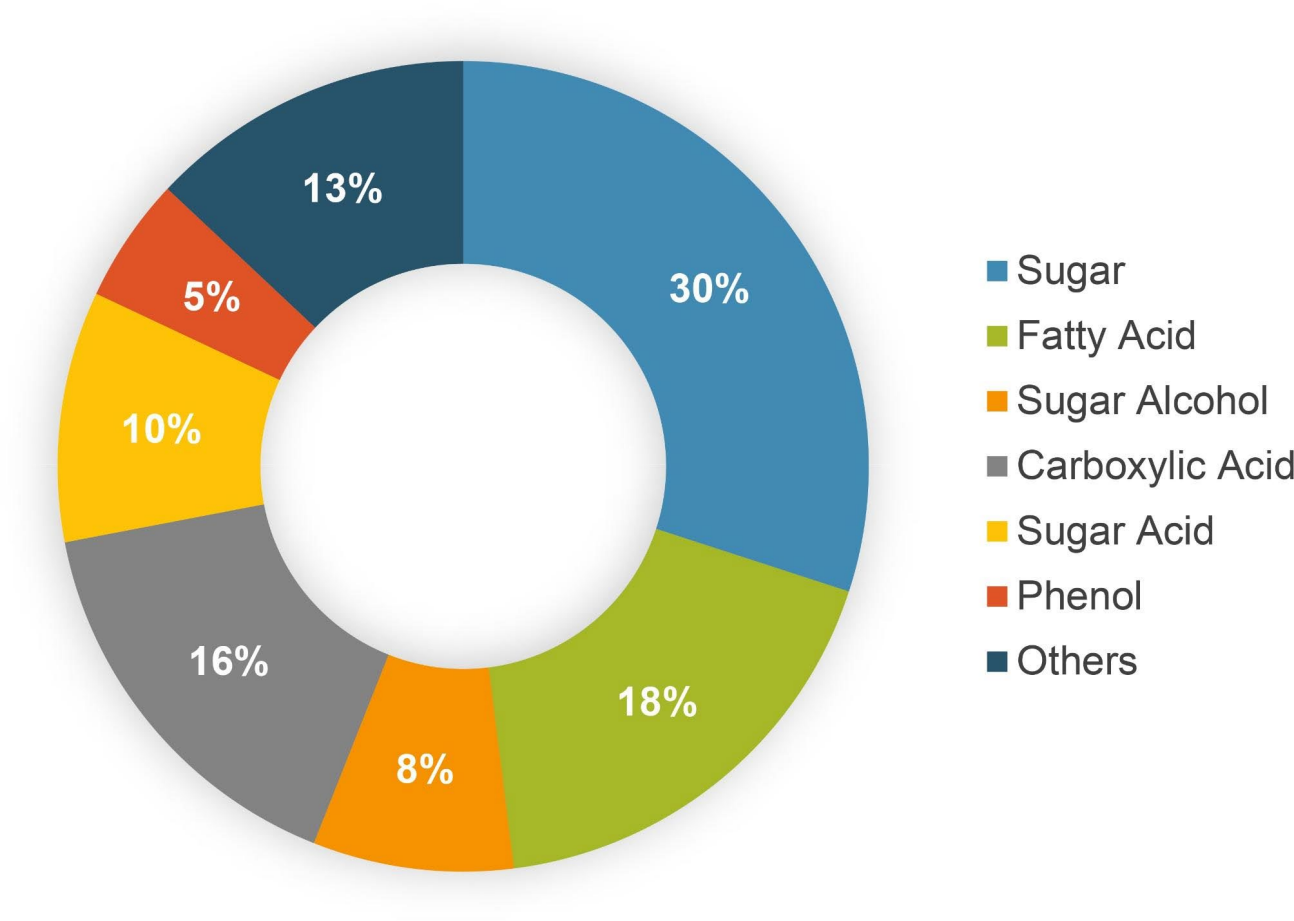
Classification of all metabolites identified by GC-MS analysis into six major groups

Morphological and microscopic study was done to study morphology of the pathogen and the infection cycle. The infection cycle of the pathogen begins with the adhesion of infectious particles (pathogen spore) to the host. The spore develops into a germ tube, that develops into an infection structure as an appressorium. An appressorium is a swollen, cylindrical organ that facilitates penetration of the pathogen into the host. Appressoria produced by some fungi, such as rust fungi, do not penetrate directly through the cuticle but through the stomata (Fig. 1). This penetration peg forms a hypha that invades and grows invasively in the host. This process leads to the development of lesions in the host and eventually to the production of further infectious particles [28]. In addition, the susceptible infected plants exhibited more severe foliar symptoms than the control sample (Fig. 1). The summary workflow of the experiment is shown in Fig. 2. To investigate the metabolites involved in plant-pathogen interactions, peanut plants were infected with *Puccinia arachidis* and analyzed by GC-MS with the NIST library. In the current study, about 61 metabolites were quantified from peanut leaf extracts (GPBD-4 and JL-24) in response to the fungal pathogen *Puccinia arachidis*. The metabolites were classified into the following biochemical groups: soluble sugars, sugar acids, fatty acids, phenols, carboxylic acids, sugar alcohols and a miscellaneous group (Fig. 3). Compared to the other metabolites, sugars and fatty acids were predominant in leaf extracts of both GPBD-4 and JL-24 genotypes.

### Alteration in the sugars and sugar acids content observed from VIP score analysis

**Fig. 4:**
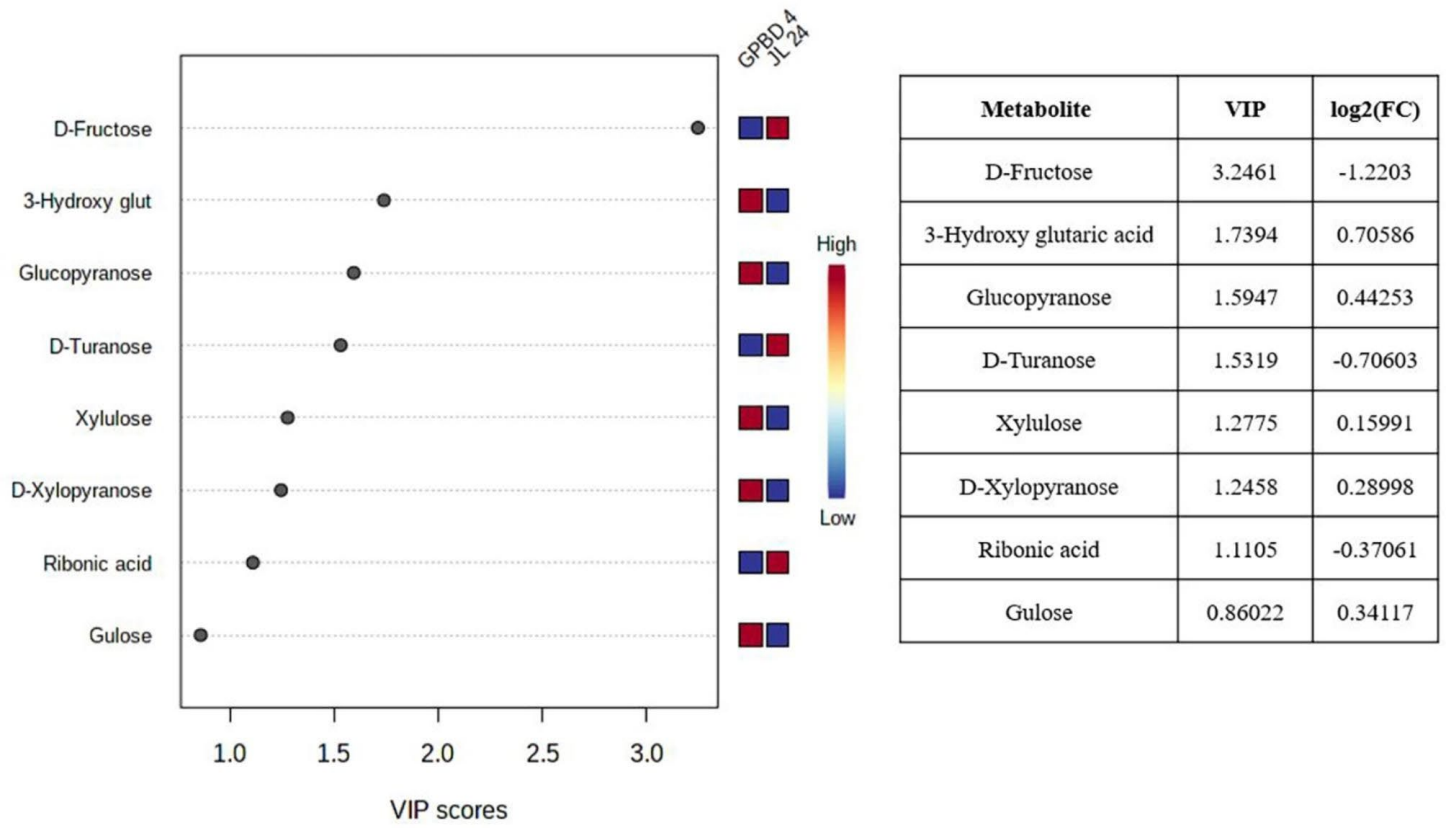
Sugar identified by Partial least squares discriminant analysis (PLS-DA) analysis of metabolites using variable importance in projection (VIP) scores in resistant (GPBD-4) and susceptible (JL-24) genotype Colored boxes indicate the relative concentrations of the corresponding metabolite in each group under current study (red, up-regulation; blue, down-regulation). Tables on the right show VIP values for Component 1 and the fold changes in the concentrations of each metabolite. Fold changes were calculated using the formula log2 (resistant/susceptible)

To determine the impact of *P. arachidis* on peanuts, we measured sugar contents in infected and un-infected leaves of GPBD-4 and JL-24 genotypes. Sugars constitutes the largest class of the total metabolites (30%) (Fig. 3). The method PLS-DA is useful for the selecting discriminative features of data useful for classifying samples. The method PLS-DA with variable importance in projection (VIP) profoundly differentiated the abundant metabolites at different stages of pathogen infection in peanut genotypes. Based on the parameter VIP > 1, a total of 20 metabolites with important variations were identified in peanut leaves. Metabolome profiling showed that the content of sugars such as D-glucopyranose, xylulose, D-xylopyranose and glucose were higher in GPBD-4 genotype as compared to JL-24 genotype. The content of fructose, turanose and ribonic acid was higher in susceptible genotype than in the resistant genotype (Fig. 4). Gulonic acid, 2-deoxy-galactose, beta-D-glucopyranose and D-turanose were higher in control samples, but the level of these sugars steadily decreases at subsequent stages of infection (Supplementary Fig. 1). The decreased sugar content suggest that it is involved in both energy production and biosynthesis of secondary metabolites as a precursor. It was reported that phenylpropanoids and flavonoids content increased during pathogen attack because they play a major role in defense [29]. D-Glucopyranose increased at IS-1 as compared to the control stage, while it rapidly decreased at subsequent infection stages in both GPBD-4 and JL-24 genotypes. At IS-2, D-xylopyranose content increase sharply compared with the control and abruptly decreased at IS-3 in GPBD-4. Some induced sugars and sugar acids such as xylulose, mannonic acid increased at IS-3 in GPBD-4, while beta-L-galactopyranose, increased at IS-3 in JL-24 genotype. The content of hexose sugars such as fructose remained low in the control leaves, but increased in the infected leaves (Supplementary Fig. 1).

### Heatmap analysis of sugar and sugar acid

**Fig. 5:**
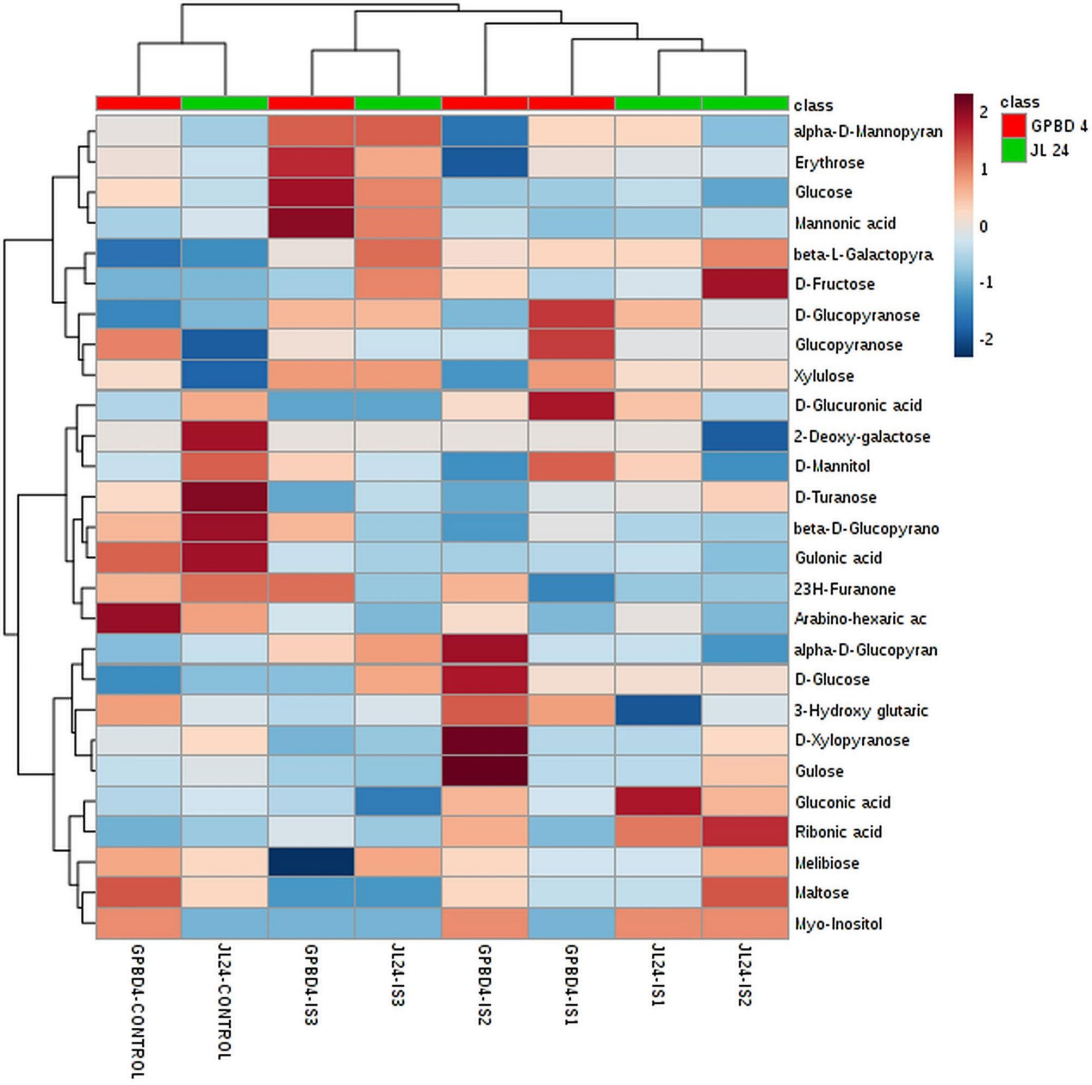
Heat map analysis showing the intensity of sugar and sugar-acid metabolites in peanut genotypes (resistant: GPBD-4 and susceptible: JL-24) at different stages. From: Control (0 DI), IS-1 (2 DAI), IS-2 (4 DAI) and IS-3 (6 DAI). IS stands for the infection stage and DAI stands for day after infection. On the log scale, brown color indicates increased sugar levels and dark blue represents decreased levels

Changes in the metabolite production were compared using a heatmap analysis. Sugars are also involved in energy production and hormonal signaling pathways of different plant physiological processes [30]. The content of sugars such as gulose, glucopyranose, xylulose, arabino-hexaric acid, melibiose and maltose was higher in control of GPBD-4 than control of JL-24. However, the levels of glucopyranose and glucuronic acid were found to be higher in resistant genotype as compared to susceptible genotype at IS-1. Sugars such as fructose and turanose content level were increased in susceptible genotype during pathogen infection (Fig. 5). A similar kind of trend was observed in pearl millet plants during downy mildew infection [31]. Few sugars such as glucopyranose and xylulose contents were higher in control and IS-1 stages, while 3-hydroxy-glutaric acid was higher at control, IS-1 and IS-2 stages of GPBD-4. 2(3 H)-Furanone, glucopyranose, glucose and xylopyranose were observed in higher concentration at IS-2 and erythrose, mannonic acid and beta-D-glucopyranose contents were observed to be higher at IS-3 (Fig. 5).

### Fatty acids, carboxylic acids, phenols and Sugar alcohols profiles by VIP score analysis

**Fig. 6:**
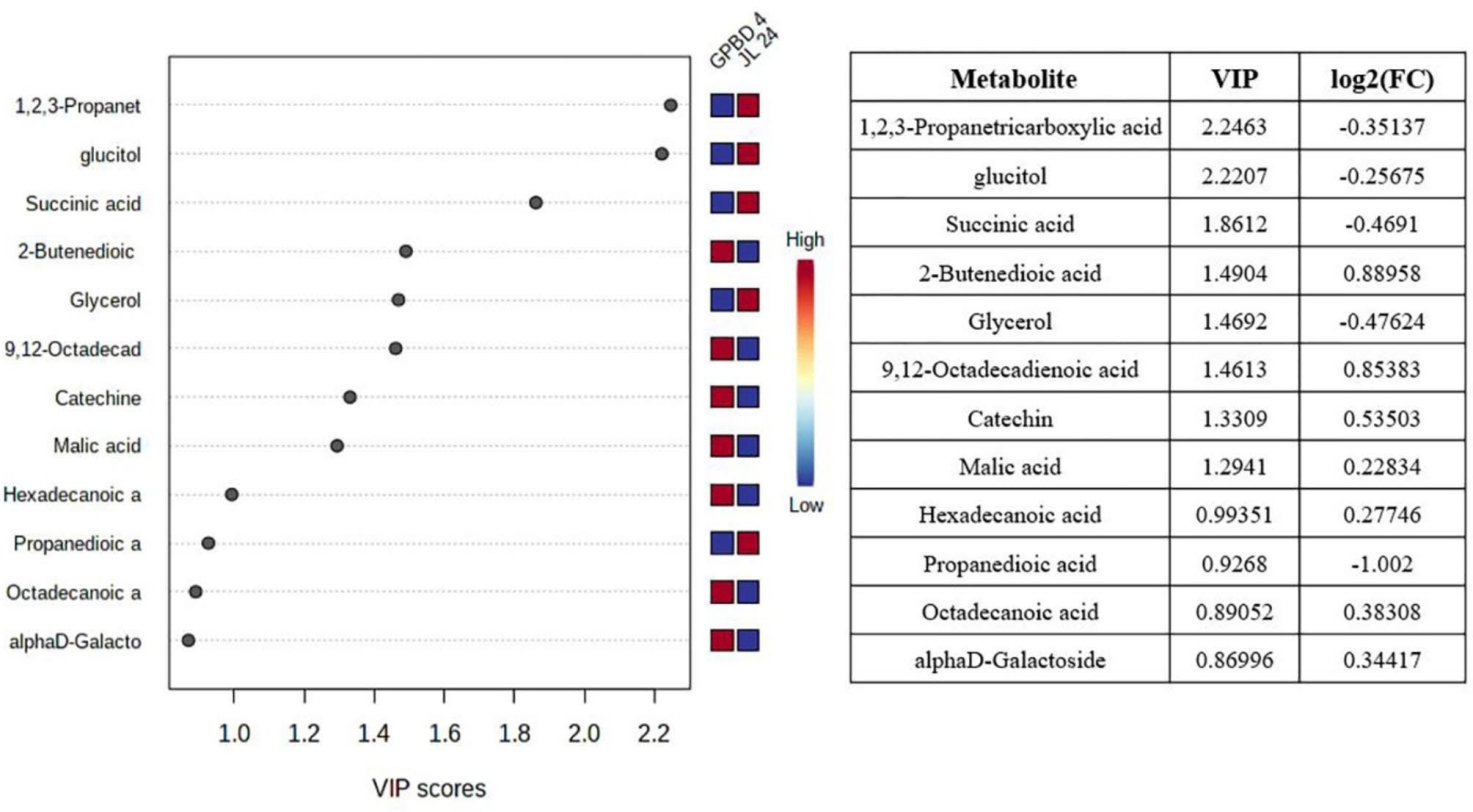
Fatty acids, phenols and organic acids identified by Partial least squares discriminant analysis (PLS-DA) analysis of metabolites using variable importance in projection (VIP) scores in resistant (GPBD-4) and susceptible (JL-24) genotype Colored boxes indicate the relative concentrations of the corresponding metabolite in each group under current study (red, up-regulation; blue, down-regulation). Tables on the right show VIP values for Component 1 and the fold changes in the concentrations of each metabolite. Fold changes were calculated using the formula log2(resistant/susceptible)

Fatty Acids (FAs) and carboxylic acids accounted for 18% and 16% of the total metabolite classes, respectively. During fungal infection, the relative content of FAs in the infected peanut leaves changed significantly. Fatty acids such as octadecanoic acid, hexadecanoic acid and 9,12-octadecenoic acid (linoleic acid) increased in the GPBD-4 (resistant) genotype as compared to JL-24 (susceptible) genotype at IS-2. Linoleic acid plays an important role in physiological effects such as germination, senescence and biosynthesis of Jasmonic acid (JA) [32]. However, the content of FAs, such as glycerol, was increased in JL-24 compared to GPBD-4 genotype (Fig. 6). Carboxylic acids play an important role in different metabolic processes by involvement in redox processes [33]. The content of propanedioic acid was higher in JL-24 genotype as compared to GPBD-4, while 2-Butenedioic acid and malic acid contents were higher in resistant genotype as compared to susceptible genotype (Fig. 6). Organic acid content, such as succinic acid was elevated at IS-3 as compared with the control stage in the susceptible genotype (Supplementary Fig. 2). However, pathogen infection of the peanutgenotypes resulted in changes in phenolic content, which accounted for 5% of the total metabolites. During pathogen infection, the resistant genotype experienced an increase in phenolic compounds such as alpha d-galactoside and alpha-d-glucopyranoside (IS-2). A similar metabolite expression was also observed in pearl millet plants during downy mildew infection. The content of flavonoid such as catechin, was found to be higher in resistant genotype then in susceptible genotype, which plays an important role in plant defense mechanism against fungal pathogen [31].

### Heatmap analysis of fatty acids, carboxylic acids, phenols and sugar alcohol

**Fig. 7:**
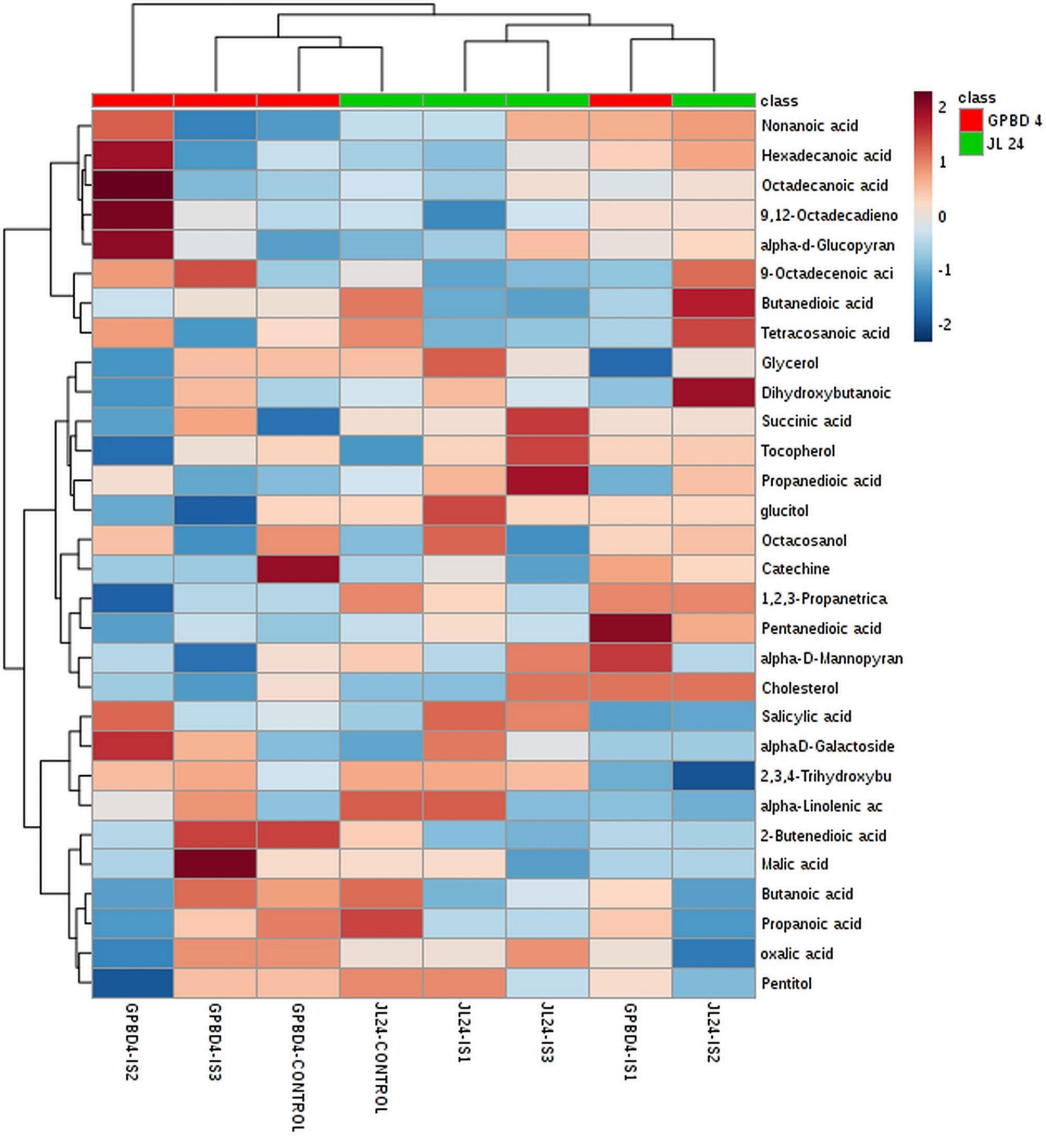
Heat map analysis showing the intensity of fatty acids, phenols and organic acids metabolites in peanut genotypes (resistant: GPBD-4 and susceptible: JL-24) at different stages. From: control (0 DI), IS-1 (2 DAI), IS-2 (4 DAI) and IS-3 (6 DAI). IS stands for the Infection stage and DAI stands for day after infection. On the log scale, brown color indicates increased sugar levels and dark blue represents decreased levels

Heat map analysis showed cluster formation between control samples of both genotypes, while IS-1 of resistant genotype showed similarity with IS-2 of susceptible genotype. There was not much difference between the profile of JL-24 (susceptible genotype) at IS-1 and IS-3, while in case of GPBD-4 (resistant genotype) metabolite profiles were found to be different at infection stages- 3 and 4 as compared to other samples (Fig. 7). Heat map of fatty acid also showed that octacosanol and cholesterol contents were higher in control of GPBD-4 (resistant genotype) than control of JL-24 (susceptible genotype). Fatty acids such as nonanoic acid, hexadecanoic acid, 9,12-octadecadienoic acid and pentanedioic acid content were higher in IS-1 and IS-2 of GPBD-4. Linolenic acid and 9-octadecenoic acid were higher in IS-3 of resistant genotype. These data indicated that a higher level of α-linolenic acid may be involved in an active plant defense response. Heat map of carboxylic acids showed that butanoic acid and propanoic acid contents were higher in IS-1 as well as IS-3, while malic acid was higher at IS-3 in GPBD-4 (resistant genotype) as compared to their control (Fig. 7). Most of the identified carboxylic acids were increased in infected leaves. The increase in succinic acids in GC/MS metabolic profiles was notable. It was increased at the IS-3 stage of GPBD-4. The oxalic acid level was increased at IS-3 as compared to IS-1 and IS-2, while gluconic acid content was increased in the IS-1 stage compared to control stage in GPBD-4 genotype (Fig. 7). Also, there was a distinguished reduction in the level of malic acid in the infected leaves at the IS-2 stage. A similar kind of trend for carboxylic acid accumulation was observed in potato sprout during *Rhizoctonia solani* infection [34]. The phenolic compounds are a major source of antioxidant activities of plants. Heat map of phenol also showed that alpha-D-mannopyranoside and alpha-d-glucopyranoside were higher in IS-1 of resistant genotype than susceptible genotype. D-galactoside was higher in IS-2 and IS-3 of resistant genotype, while salicylic acid was higher in only IS-2 of GPBD-4 as compared to susceptible genotype. A similar pattern was observed in the soybean plant for salicylic acid at 24 h after the inoculation of *B. japonicum* [35]. Sugar alcohol such as pentitol content was observed to be higher at IS-3 of GPBD-4 (resistant genotype) as compared to IS-1 and IS-2. D-mannitol was found to be higher at IS-1, IS-2 and IS-3 stages of GPBD-4 (resistant genotype).

**Fig. 8:**
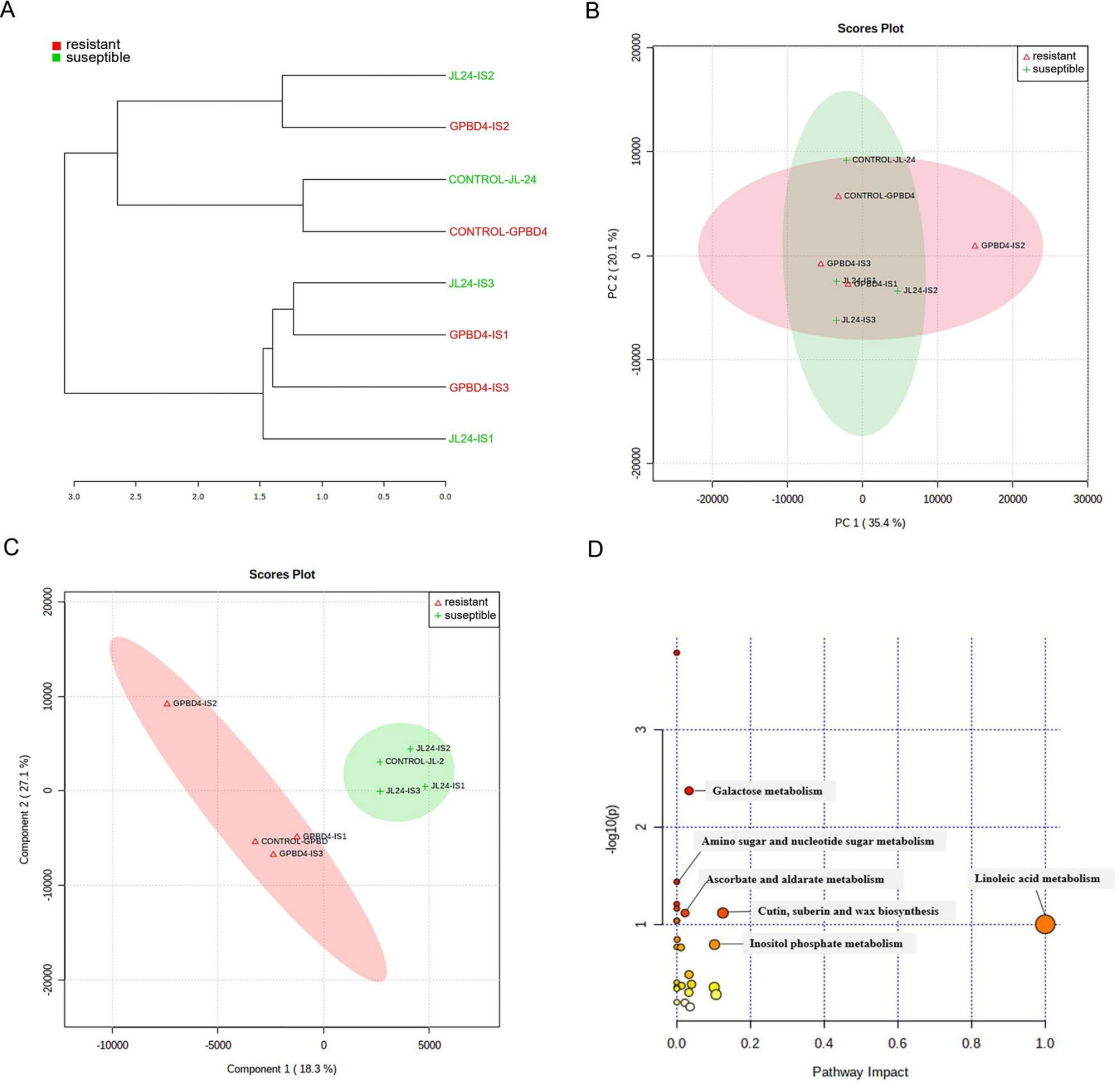
**A**. The dendrogram analysis of peanut genotypes at different stages *P. arachidis* fungal infection **B**. PCA score plot analysis of peanut genotypes at different infection stages **C**. PLS-DA 2D score plot analysis of peanut genotypes at different infection stages **D**. Metabolomic Pathway Analysis (MetPA) as generated by MetaboAnalyst software

Based on the metabolite pattern, 2 clusters were formed in which cluster-I comprised control and IS-2 stages of both genotypes JL-24 and GPBD-4 (Fig. 8a), whereas, in cluster-II, IS-1 and IS-3 stages of both genotypes JL-24 and GPBD-4 were grouped. Cluster I is subdivided into two sub-clusters. In sub-cluster A consists of JL-24 IS-2 and GPBD-4 IS-2, and sub-cluster B contains control JL-24 and control GPBD-4. Cluster II was subdivided into two sub-clusters. In sub-cluster A contained JL-24 IS-3, GPBD-4 IS-1 and GPBD-4 IS-3, sub-cluster B had only JL-24 IS-1. The metabolite pattern of the control samples in both genotypes almost remained the same, while JL-24 IS-2 and GPBD-4 IS-2 were also put together in the same cluster formation which is reflected by the dendrogram.

### PLSDA, PCA and pathway analysis of the whole metabolome

A distinguished metabolite pattern was observed in the leaf samples taken from resistant and susceptible genotypes. According to the PCA (Principal Component Analysis), 7 principal components (PCs) were gained from the comparison between resistant and susceptible peanut genotypes. From the diverse multivariate handling techniques, PCA was used to identify metabolic changes in control and *Puccinia arachidis* infected peanut GPBD-4 and JL-24 plants. The separation was observed between infected and control samples of both genotypes by the PCA score plot (Fig. 8b). Data analysis by the PCA showed that the first two components explained 55.5% of the overall variance. The first principal component (PC1) alone revealed 35.4% of the total variance present within the data, while the second principal component (PC2) contributed to 20.1% of the total variance (Fig. 8b). To confirm PCA results, we performed a supervised PLS-DA (Partial Least Square Discriminant Analysis) method which was applied to identify common metabolic changes in plants after fungal infection. All the metabolites’ data evaluated by the PLS-DA score plot showed two components explaining 45.4% of the overall variance. The first component revealed 18.3% of the total variation, while the second component contributed to 27.1% of the total variation (Fig. 8c).

A detailed analysis of the relevant pathways and networks affected by rust infection in peanut plant was performed by the web-based tool MetPA (Metabolic Pathway Analysis) which combines results from a powerful pathway enrichment and topology analysis. The results from the current investigation showed that upon biotic stress, major defense-related metabolites such as linoleic acid is up-regulated as compared to the control, clearly indicating the activation of a tolerance mechanism to protect plant against pathogen. A few important metabolic pathways were found related to fatty acid, sugar, sugar alcohol and phytoalexin compounds metabolism including: (i) galactose metabolism; (ii) cutin, suberin and wax metabolism; (iii) Ascorbate and aldarate metabolism; (iv) inositol metabolism; (v) linoleic acid metabolism (Fig. 8d).

### Enzyme activity (catalase, peroxidase and polyphenol oxidase)

**Fig. 9:**
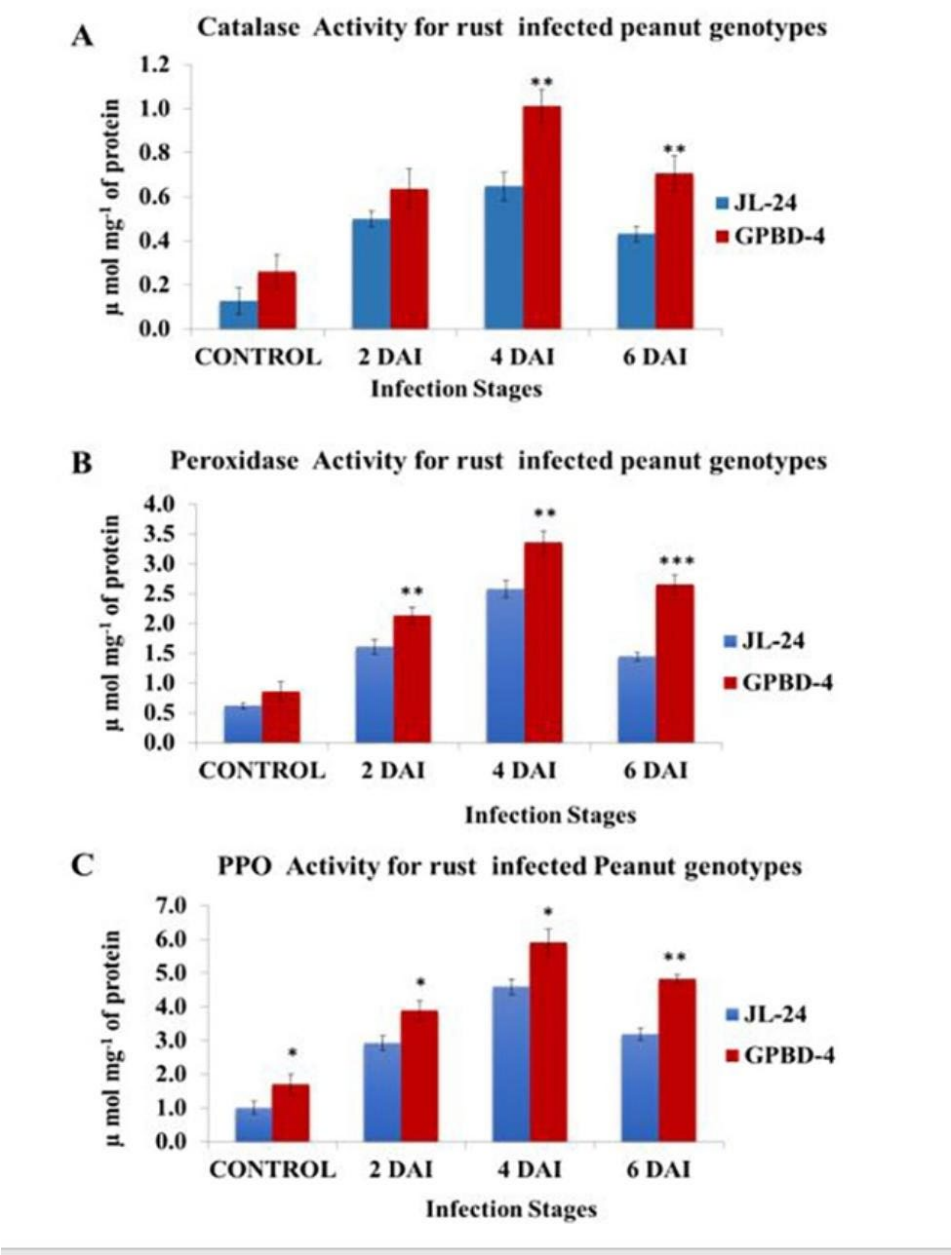
Effects of rust infection by *Puccinia arachidis* on enzyme activities in resistant (GPBD-4) and susceptible (JL-24) genotypes of peanut. (**A**) catalase enzyme activity, (**B**) peroxidase enzyme activity and (**C**) Polyphenol oxidase enzyme activity. The pre-infection samples were designated as control, 2 DAI, 4DAI, 6 DAI (DAI stands for Days after infection). (*: p-value < 0.1, **: p-value < 0.01 and ***: p-value < 0.001, when compared with JL-24 (S) variety)

Selected enzyme activities were analysed to validate the data and observation from metabolomic study. Catalase (CAT) and Peroxidase (POD) are the most important enzymes involved in the regulation of intracellular levels of H_2_O_2_, leading to stress tolerance. Different enzyme activities such as CAT, POD and Poly Phenol Oxidase (PPO) were found to be higher in resistant genotype (GPBD-4) as compared to susceptible genotype (JL-24) (Fig. 9). In rust infection, higher induction of CAT was observed in GPBD-4 in IS-1 and IS-2, while it was sharply reduced in IS-3 as compared to their respective control. In the case of JL-24, CAT was increased in all stages but in IS-3 it showed a slow decline (Fig. 9A). A similar pattern was also observed in POD (Fig. 9B) and PPO enzyme activities (Fig. 9c). These findings indicate that the up-regulation of these compounds is likely to reinforce responses of plant resistance against pathogen attack.

## Discussion

In this study, the metabolite changes were analysed in the resistant and susceptible genotypes of peanut plants in response to fungal pathogen *Puccinia arachidis*. However, studies on metabolic profiling of peanut plants against *P. arachidis* have been limited. There is also very limited information on changes in metabolome profile of peanut plants with respect to other fungal infections [15]. This study contributes to understand plant-pathogen interaction by providing information related to the metabolomic status of plant cells under biotic stress. The plant defense mechanism involves the production of oxidative enzymes involved in the formation of phenylpropanoid and antimicrobial metabolites (flavonoid), that affect pathogen growth [31]. Plant metabolites such as sugars, phenols, sugar alcohols, organic acids and fatty acids are involved in biotic stress responses. During plant-pathogen interaction, accumulation of metabolites indicated the activation of the induced systemic resistance (ISR) system, which involves enzyme synthesis and deposition of lignin in the cell wall [36, 37]. Few defense related resistant genes such as, β-glucosidase, chitinase, Thaumatin, cytochrome P450, Glutathione S-transferase and Terpene synthase were highly up-regulated (> 8.0-fold) in resistant genotype during *P. arachidis* infection. These highly expressed genes were expected to play a crucial role in the defense mechanisms during fungal pathogen infection in *A. hypogaea*, which helped it to sustain under biotic stress [38].

The reports on the genes and metabolic regulation in response to *P. arachidis* in peanut are very limited. Though few resistant genes such as, NBS leucine- rich repeats, pathogenesis related protein 10 and glucosyl hydrolase protein have been reported in peanut. Two RAPD markers, two SSR markers and 12 Quantitative trait loci (QTL) were identified for rust resistance in peanut plant. One important resistance related SSR marker was found on linkage group A03 [39], while in our study we also observed defense related genes on A03 chromosome (F box protein, protein kinase, cytochrome p450, NBS-LRR) [38]. In this study different sugar and fatty acid metabolites and hydrolase enzymes (for fungal cell-wall degradation, plant lignification, activation of hypersensitive response) were produced in resistant variety during rust infection for defense mechanism. Such metabolites and molecular markers are used to study genetic diversity, genetic mapping and for development of plant variety.

The accumulation of D-fructose and D-turanose was reduced to IS-3 in peanut leaves. This reduction in carbohydrate content after pathogen infection suggests involvement in energy production and formation of secondary metabolites, including phenolic biosynthesis, which are important compounds for plant defense [40]. Such a reduction in fructose content was observed in potato sprout during *Rhizoctonia solani* fungal infection [34]. Carbohydrates may also act as antioxidants and signaling molecules [14]. The content of glucose and xylulose increased significantly in peanut during infection with *P. arachidis* compared to the control sample. The accumulation of glucose suggests its role in plant defense mechanism during host-pathogen interactions [41]. In the host plant maize, hexose content increased after infection with the *Ustilago maydis* [42]. Sugars play an important role in stress resistance as they are the precursors for the synthesis of phytoalexins and phenols, which suppress the activity of cellulolytic enzymes important for pathogenesis [43]. Sugar alcohols such as mannitol and myo-inositol were enriched in the biotic stress [44]. Mannitol and probably other sugar alcohols play an important role in plant pathogen resistance by protecting against reactive oxygen species (ROS) [45]. After pathogen infection, the content of myo-inositol was increased in JL-24 at IS-1 and IS-2, while it decreased in GPBD-4. A similar trend was observed in pearl millet plants during downy mildew infection [31].

Fatty acids play an important role in plant germination, senescence and JA synthesis [32]. Linolenic acid is a precursor of JA, which is involved in plant defense mechanisms against pathogen attack [46]. There was an accumulation of linolenic acid in potato leaves during *Pseudomonas syringae* infection, which prevented the fungal growth [47]. Moreover, Octadecadienoic acid (Linoleic acid) is a substrate of lipoxygenase enzyme which catalyzes the conversion of linoleic acid to hydroperoxides that are in turn converted to oxylipins which is a precursor of JA. Oxophytodienoic acid reductase gene expression was upregulated (Aradu.U86RL, 1.43 fold) in GPBD-4 resistant genotype during rust infection [38]. In our study, 9, 12-octadecadienoic acid (precursor of JA) content was increased in resistant genotype during *Puccinia arachidis* infection. A similar pattern was also observed in potato plants during *Phytophthora infestans* infection [48]. During the pathogen attack, linoleic acid content increased in avocado plants, which shows its antifungal activity [49]. Different PR-metabolites such as butanedioic acid, 9,12-octadecadienoic acid, D-xylopyranose and hexadecanoic acid were increased in resistant genotype after pathogen inoculation. Phenolics compounds are involved in the prevention of fungal growth inside plant tissue by inhibiting fungal mycelia growth and spore germination [50]. Phenolics and tocopherol level was increased in potato sprout due to *Rhizoctonia solani* infection [34]. In our study, salicylic acid level was increased in infected plants for defense as compared to control plants. The susceptibility of transgenic tobacco to diseases increased with the suppression of the level of phenolic compounds [51].

Propanoic acid is an intermediate in the salicylic acid biosynthesis pathway, which is an important signaling molecule in the plant defense system [52]. In this study increased level of butanoic and propanoic acids was observed in resistant genotype as compared to control plants. A similar pattern was also found in potato plants during *Phytophthora infestans* infection [5]. Carboxylic acids play many important roles in plant growth and pathogenesis. Increased level of oxalic acid in infected leaves is indicative of its implication in plant-pathogen interactions. In our experiments, malic and oxalic acid content was increased in resistant genotype at IS-3 as compared to control samples. Oxalic acid is catalyzed by oxalate oxidase (OXO) resulting in the production of H_2_O_2,_ which leads to hypersensitive response (HR) in plants. Such fungitoxicity was recorded in the case of potato sprout as well as sunflower during pathogen infection [34, 53]. Malic acid is involved in the central metabolic pathway and acts as an osmolyte during stomatal responses [33]. Malate and succinate contents were also found to be higher in the roots of a soybean genotype during *F. tucumaniae* infection [54].

In the case of susceptible genotype, gluconic acid content was increased to IS-1 compared with the control stage. This increased gluconic acid content indicates activation of the pentose phosphate pathway to produce basic nucleotide components such as NADPH and pentoses. A similar trend for gluconic acid was observed in potato plants during pathogen infection [34]. The 2,3,4-trihydroxybutyric acid was also considered as a defense-related metabolite in peanut, which showed a significant increase at IS-3 compared to the control stage. The up-regulation of this organic acid was also found in potato leaves during inoculation with *Phytophthora infestans* [48]. Flavonoids are frequently detected as biomarkers of fungi infected plants. A previous study reported flavonoid accumulation in peanut plants during infection with *S. rolfsii* [15]. In our study, catechin content was found to be higher in the resistant (GPBD-4) genotype compared to the susceptible genotype.

During the plant-pathogen interaction, the enzymes CAT, POD and PPO are involved in the detoxification of ROS, such as H_2_O_2_, leading to the activation of a hypersensitive response [55]. The higher activity of CAT enzymes in GPBD-4 (resistant genotypes) prevents the accumulation of H_2_O_2_ in plant cells to toxic levels [43]. PPO enzymes play an important role in the oxidation of polyphenols and lignification of plant cells and are involved in plant’s defense mechanisms against biotic stress [50]. The study revealed higher activity of POX enzymes in resistant genotypes compared to susceptible ones. The highest activity of all three enzymes was observed four days after inoculation, after which the activities of these enzymes steadily decreased. Peroxidases are also important PR proteins involved in lignin biosynthesis and production of active oxygen species which help to prevent pathogen spread and viability [56]. Cowpea plants inoculated with *S. rolfsii* fungi showed increased POD and PPO enzyme activities after inoculation, but these decreased after 5 days of inoculation [57]. To investigate the general metabolic effects of biotic stress, a comparison of control and infected samples was also performed using multivariate analysis (pattern recognition). PCA analysis of metabolic profile showed that the control samples differed from the remaining infected samples of both genotypes at different stages of infection. Analysis of PLS-DA distinguished the resistant genotype from the susceptible genotype based on the pattern of their metabolites.

Metabolomic profiling provides a better understanding of the biochemical status of any organism during biotic stress. The increased levels of fatty acids and phenolic compounds in plant-fungal interaction support their role in plant resistance. This study concludes with the biological role of different metabolites such as fatty acids, phenolics, carboxylic acid and sugars in plant defense mechanisms. The metabolites found in abundance were associated with a plant defense mechanism. Such metabolites may be considered as resistance biomarkers for the varietal selection of peanut plants against rust disease.

### Electronic supplementary material

Below is the link to the electronic supplementary material.

**Supplementary Figure 1**. Sugar identified by PLS-DA of metabolites using VIP scores at control and three infection stages (IS1, IS2, IS3) in both genotypes. Colored boxes indicate the relative concentrations of the corresponding metabolite at different stages of infection (red: up-regulation; blue: down-regulation). **Supplementary Figure 2**. Fatty acids, phenols and organic acids identified by PLS-DA of metabolites using VIP scores at control and three infection stages (IS1, IS2, IS3) in both genotypes. Colored boxes indicate the relative concentrations of the corresponding metabolite at different stages of infection (red: up-regulation; blue: down-regulation).

## Data Availability

the original contributions presented in the study are included in the article/Supplementary Material; further inquiries can be directed to the corresponding authors.

## List of abbreviations

HR: Hypersensitive reaction
GC- MS: Gas Chromatography-Mass Spectrometry
PLS-DA: Partial least square-discriminant analysis
DAI: Day after inoculation
LLS: Late leaf spot
ELS: Early leaf spot
LC-MS: Liquid Chromatography-Mass Spectrometry
PCA: Principal components analysis
MetPA: Metabolomic Pathway Analysis
