# Supplementary material for: Metabolic profiles of peanut (Arachis hypogaea L.) in response to Puccinia arachidis fungal infection
Source: BMC Genomics. 2023 Oct 23;24:630. doi: 10.1186/s12864-023-09725-3 (PMC10591357; doi:10.1186/s12864-023-09725-3)
Supplement: Supplementary file 1 — Supplementary Figure 1. Sugar identified by PLS-DA of metabolites using VIP scores at control and three infection stages (IS1, IS2, IS3) in both genotypes. Colored boxes indicate the relative concentrations of the corresponding metabolite at different stages of infection (red: up-regulation; blue: down-regulation). Supplementary Figure 2. Fatty acids, phenols and organic acids identified by PLS-DA of metabolites using VIP scores at control and three infection stages (IS1, IS2, IS3) in both genotypes. Colored boxes indicate the relative concentrations of the corresponding metabolite at different stages of infection (red: up-regulation; blue: down-regulation). [file 12864_2023_9725_MOESM1_ESM.docx]

**Supplementary Figure 1.**


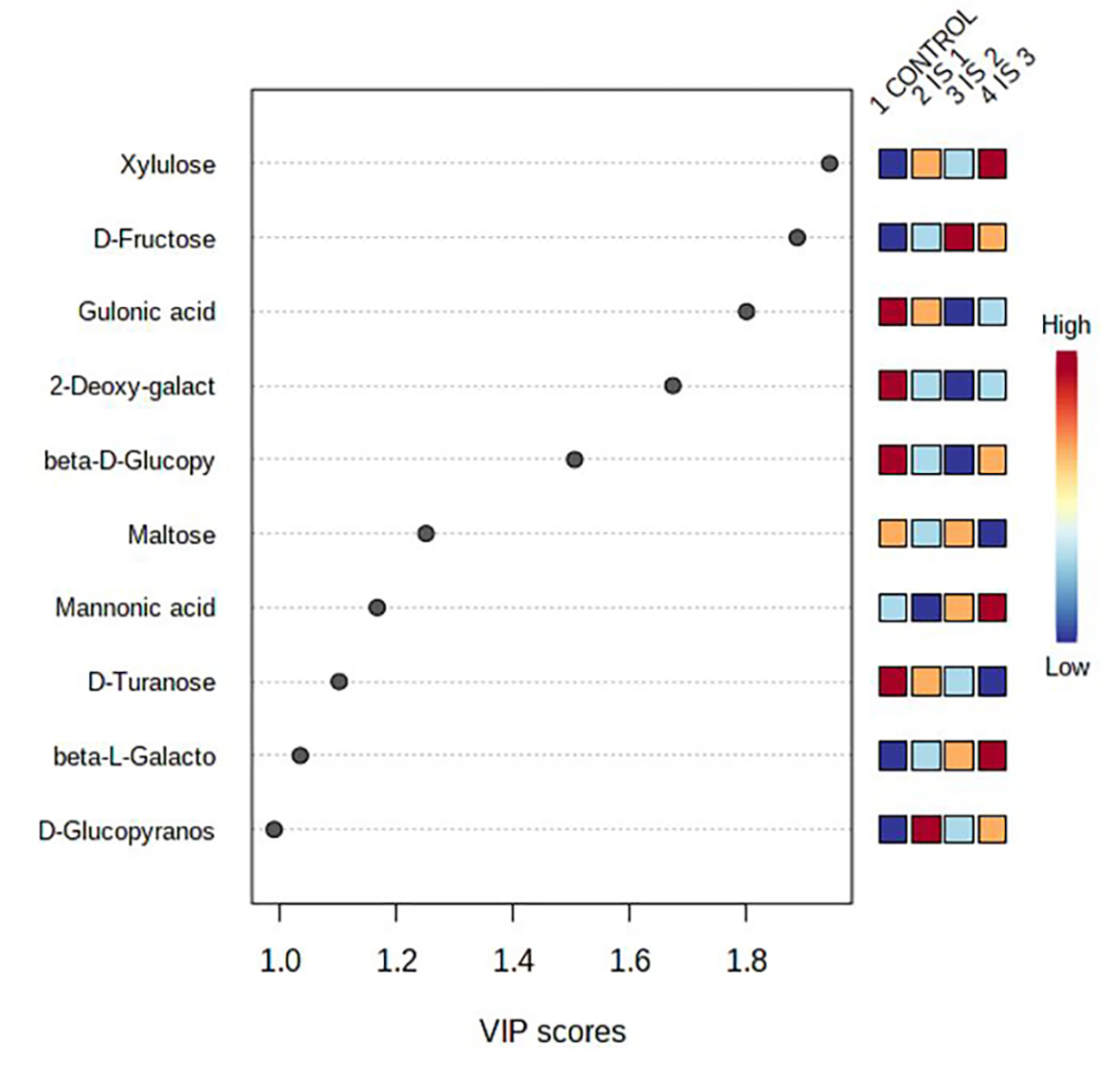


Supplementary Figure 1. Sugar identified by PLS-DA of metabolites using VIP scores at control and three infection stages (IS1, IS2, IS3) in both genotypes. Colored boxes indicate the relative concentrations of the corresponding metabolite at different stages of infection (red: up-regulation; blue: down-regulation).

**Supplementary Figure 2.**


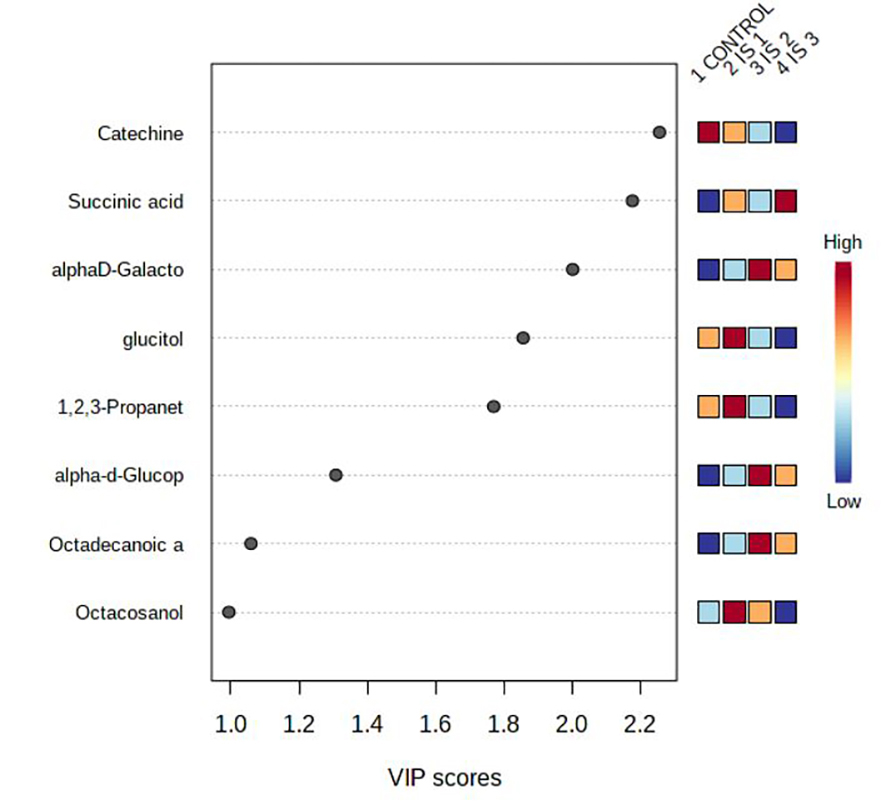


Supplementary Figure 2. Fatty acids, phenols and organic acids identified by PLS-DA of metabolites using VIP scores at control and three infection stages (IS1, IS2, IS3) in both genotypes. Colored boxes indicate the relative concentrations of the corresponding metabolite at different stages of infection (red: up-regulation; blue: down-regulation).
